# Isopsoralen ameliorates rheumatoid arthritis by targeting MIF

**DOI:** 10.1186/s13075-021-02619-3

**Published:** 2021-09-17

**Authors:** Yi Han, Jinguang Wang, Shufeng Li, Yi Li, Yongli Zhang, Ruojia Zhang, Yuang Zhang, Huancai Fan, Haojun Shi, Jihong Pan, Guanhua Song, Luna Ge, Lin Wang

**Affiliations:** 1 China Academy of Chinese Medical Sciences, Guang’anmen Hospital, Beijing, China; 2 Department of Orthopedics, Dezhou People’s Hospital, Dezhou, Shandong China; 3 Department of Orthopedic Surgery, The First Affiliated Hospital of Shandong First Medical University, Jinan, China; 4 Department of Joint Surgery, Shandong Provincial Hospital Affiliated to Shandong University, Jinan, China; 5 Biomedical Sciences College & Shandong Medicinal Biotechnology Centre, Key lab for Biotech-Drugs of National Health Commission, Key Lab for Rare & Uncommon Diseases of Shandong Province, Shandong First Medical University & Shandong Academy of Medical Sciences, Jinan, China; 6 Department of Rheumatology and Autoimmunology, The First Affiliated Hospital of Shandong First Medical University, Jinan, China; 7 Biomedical Sciences College & Shandong Medicinal Biotechnology Center, Key lab for Biotech-Drugs of National Health Commission, Key Lab for Rare & Uncommon Diseases of Shandong Province, Shandong First Medical University & Shandong Academy of Medical Sciences, #18877, Jingshi Road, Jinan, 250062 China; 8 The Second Clinical Medical College, Henan University of Chinese Medicine, Jinan, China; 9 Institute of Basic Medicine, Shandong First Medical University & Shandong Academy of Medical Sciences, Jinan, China

**Keywords:** Rheumatoid arthritis, Isopsoralen, MIF, Collagen-induced arthritis, RA FLSs

## Abstract

**Background:**

Isopsoralen (IPRN), one of the active ingredients of *Psoralea corylifolia* Linn, has anti-inflammatory properties. We attempted to investigate the inhibitory effects of IPRN on rheumatoid arthritis (RA) and characterize its potential mechanism.

**Methods:**

RA fibroblast-like synoviocytes (FLSs) and mice with collagen-induced arthritis (CIA) were used as in vitro and in vivo models to analyze the antiarthritic effect of IPRN. Histological analysis of the inflamed joints from mice with CIA was performed using microcomputed tomography (micro-CT) and hematoxylin-eosin (HE) staining. RNA sequencing (RNA-Seq), network pharmacology analysis, molecular docking, drug affinity responsive target stability (DARTS) assay, and cellular thermal shift assay (CETSA) were performed to evaluate the targets of IPRN.

**Results:**

IPRN ameliorated the inflammatory phenotype of RA FLSs by inhibiting their cytokine production, migration, invasion, and proangiogenic ability. IPRN also significantly reduced the severity of CIA in mice by decreasing paw thickness, arthritis score, bone damage, and serum inflammatory cytokine levels. A mechanistic study demonstrated that macrophage migration inhibitory factor (MIF), a key protein in the inflammatory process, was the specific target by which IPRN exerted its anti-inflammatory effects in RA FLSs.

**Conclusion:**

Our study demonstrates the antiarthritic effect of IPRN, which suggests the therapeutic potential of IPRN in RA.

**Supplementary Information:**

The online version contains supplementary material available at 10.1186/s13075-021-02619-3.

## Introduction

Rheumatoid arthritis (RA) is a chronic, autoimmune inflammatory disease characterized by synovial hyperplasia and joint destruction [1]. Persistent inflammation caused by the excessive production of inflammatory cytokines, such as tumor necrosis factor-α (TNF-α), interleukin (IL)-1, IL-6, IL-17, and macrophage-stimulating factor (M-CSF), by fibroblast-like synoviocytes (FLSs), as well as recruited macrophages and immune cells, promotes the formation of pannus and consequently destroys the articular cartilage and bone [2, 3]. RA FLSs exhibit tumor-like properties, including enhanced proliferation, prolonged survival, and apoptosis resistance [4, 5]. In addition, RA FLSs can secrete many proinflammatory factors, thereby recruiting inflammatory cells (including macrophages and lymphocytes), inhibiting their apoptosis [6, 7], and ultimately promoting local and systemic inflammation. More importantly, RA FLSs produce excessive amounts of matrix metalloproteinases (MMPs), which directly invade and destroy the adjacent articular cartilage [8]. Thus, targeting RA FLSs represents a potential strategy for RA treatment.

Traditional Chinese medicine (TCM) has long been applied to treat chronic inflammatory diseases and shows efficacy in RA [9, 10]. In recent years, numerous studies have been carried out to study the anti-RA effect of ingredients from TCM [11]. To date, botanical medicines, such as sinomenine [12], tripterygium glycosides [13], and total glucosides of peony [14], have been developed and applied as alternative treatments for RA. Isopsoralen (IPRN, 2H-Furo[2,3-H] chromen-2-one, CAS Number: 523-50-2, Fig 3b), one of the main ingredients in the seeds of the *Psoralea corylifolia Linn* plant, has been reported to exert anti-inflammatory effects in inflammatory respiratory and neurodegenerative diseases [15, 16]. In this study, we aimed to investigate the therapeutic potential of IPRN in RA and the molecular mechanism.

## Methods

### Patients and preparation of RA FLSs

Synovial tissues were obtained from 10 patients who underwent joint replacement surgery at Shandong Provincial Hospital. All the patients were diagnosed according to the 1987 American College of Rheumatology (ACR) revised classification criteria [17] or the European League against Rheumatology (EULAR) classification criteria for RA [18]. This study was approved by the Medical Ethics Committee of the Institutional Review Board of Shandong Medicinal Biotechnology Center (SMBC2020-07), and all the patients signed informed consent forms.

RA FLSs were isolated by the modified tissue culture method we previously described [19]. The cells were cultured in Dulbecco’s modified Eagle’s medium (DMEM, Hyclone, Logan, UT, USA) supplemented with 10% fetal bovine serum (FBS) and 1% antibiotics. RA FLSs from passages 4~6 were used in our experiments.

### Migration, invasion, and tube formation assays

RA FLSs were pretreated with or without TNF-α (Novoprotein, Shanghai, China) in the presence or absence of IPRN (MedChemExpress, Monmouth Junction, NJ, USA) for 24 h. The cell supernatants were collected as a conditioned medium and cultured with HUVECs seeded in 48-well plates for tube formation assays. Migration and invasion assays were performed using Transwell chambers with 8-μm pores (Corning, Tewksbury, MA, USA) as previously described [19]. For the invasion assay, Matrigel (Corning, Tewksbury, MA, USA) was precoated on the membrane of the upper chamber, and the cells were seeded as in the migration assay and allowed to invade for 24 h. For the tube formation assay, Matrigel was precoated on 48-well plates. The data are presented as the mean number of migrated cells in six randomly chosen fields.

### Quantitative real-time PCR (qRT-PCR) and enzyme-linked immunosorbent assay (ELISA)

Total RNA was isolated from RA FLSs using TRIzol Reagent (Invitrogen, Carlsbad, CA, USA) according to the manufacturer’s protocol. RNA was reverse-transcribed using a ReverTra Ace qPCR RT Kit (Toyobo, Shanghai, China), and qRT-PCR was performed using a Light-Cycler 480 (Roche, Basel, Switzerland). All the primers were synthesized by Beijing Genomics Institute (BGI, Beijing, China), and the sequences of all the primers used in this study are listed in Supplied Table 1. Relative mRNA levels were measured using the 2^-Δ^Cycle Threshold (2^-ΔCT^) method [20]. Three independent experiments were performed, and each reaction was repeated three times.

The levels of IL-1β, IL-6, cartilage oligomeric matrix protein (COMP), and IL-10 in the supernatant were determined using commercial ELISA kits (Multi Sciences (Lianke) Biotech Co., Ltd., Hangzhou, China) according to the manufacturer’s instructions.

### Small interfering RNA transfection and RNA sequencing

The negative control (NC) siRNA and siRNA targeting macrophage migration inhibitory factor (MIF) were synthesized by Ruibo Co., Ltd. (Guangzhou, China). siRNA targeting MIF or NC siRNA was transfected into RA FLSs using transfection reagent (PolyPlus Transfection, Strasbourg, France) according to the manufacturer’s instructions. Total RNA was extracted by TRIzol to conduct RT-qPCR or cDNA library construction for RNA transcriptome sequencing by LC-BIO Technologies Co., Ltd. (Hangzhou, China).

### Drug affinity responsive target stability (DARTS) assay, cellular thermal shift assay (CETSA), and Western blotting analysis

DARTS assay [21], CETSA assay [22], and Western blotting analysis were conducted as previously described. Protein samples were separated by SDS-PAGE and electrophoretically transferred onto 0.45-μm polyvinylidene fluoride (PVDF) membranes (Millipore, Bedford, MA, USA). The PVDF membranes were blocked and then incubated at 4°C overnight with primary antibodies against MIF (Abclonal, Wuhan, China) and glyceraldehyde-3-phosphate dehydrogenase (GAPDH) (Proteintech, Wuhan, China). Subsequently, the membranes were washed three times with tris-buffered saline with Tween (TBST) and incubated with HRP-conjugated AffiniPure goat anti-rabbit IgG (H+L) (Proteintech, Wuhan, China) for 1 h at room temperature followed by three washes with TBST. The blots were visualized using an enhanced chemiluminescent substrate (ECL) kit (Vazyme, Nanjing, China). The protein bands were quantified using ImageJ software 2.0 (National Institutes of Health, Bethesda, Maryland, USA) and were normalized to the density of the respective GAPDH band.

### Network pharmacology analysis, molecular docking, and MIF tautomerase assay

Network pharmacology was used to predict targets as previously described [23]. PharmMapper (http://lilab-ecust.cn/pharmmapper/), STITCH (http://stitch.embl.de/), and TargetNet (http://targetnet.scbdd.com/calcnet/index/) were used to predict the targets of IPRN. Duplicates were deleted after combining the targets of IPRN. Targets relevant to rheumatoid arthritis were identified by searching the NCBI gene database with the following keyword: “rheumatoid arthritis.” Molecular docking was performed on the Yinfo Cloud Computing Platform (https://cloud.yinfotek.com). l-Dopachrome substrate was prepared through oxidation of l-3,4-dihydroxyphenylalanine methyl ester (Aladdin, Shanghai, China) with sodium periodate (Aladdin, Shanghai, China) [24]. Recombinant MIF (Abcam, Cambridge, MA, USA) (100 ng/mL) was incubated with IPRN or the MIF inhibitor ISO-1 (Selleck, Shanghai, China) at the indicated final concentrations [25]. The decrease in absorbance was measured at 475 nm every 10 s for 6 min.

### CIA model

All the animal studies were approved by the Institutional Animal Care and Use Committee of Shandong First Medical University & Shandong Academy of Medical Sciences. Eight-week-old male DBA/1J mice purchased from Vital River Laboratory Animal Technology (Beijing, China) were raised under specific pathogen-free conditions and were given free access to a standard diet and water. CIA models were established as previously described [19]. Briefly, CIA was induced in mice by injecting 2 mg/mL bovine type II collagen (Chondrex, Redmond, WA, USA) and complete Freud’s adjuvant (Chondrex, Redmond, WA, USA) in a 1:1 emulsion (200 μL) at the base of the tail. Booster immunization with a 1:1 emulsion of bovine type II collagen and incomplete Freud’s adjuvant (200 μL) was administered 21 days after the first immunization. In the therapeutic treatment study, when the arthritis score reached 6, the mice were randomly divided into groups. IPRN or vehicle (9 mice per group) was administered by intraperitoneal injection into the mice twice a day beginning on the day the mice were divided into groups. The progression of arthritis was continuously monitored after the first immunization by assessing the arthritis score (the sum of scores on a 0–4 scale for all 4 limbs, based on the swelling of the limbs, for a total possible score of 16 for each mouse) using previously described methods [26]. The arthritis score for each mouse was defined as the sum of the scores of all four paws. In each category, the joints were independently evaluated in a blinded manner. In addition, the thickness of the arthritic hind paws was measured daily with microcalipers.

### Micro-computed tomography (micro-CT)

Micro-CT (QuantumGX, PerkinElmer, United States) was performed on the hind paws of mice with CIA with the following parameters: exposure time of 14 min at 90 KV and 88 mA with a resolution of 2 μm and field-of-view 12.8 mm× 12.8 mm. The parameters of the trabecular bone, including bone volume to bone volume fraction (BV/TV, %), trabecular thickness (Tb.Th, μm), trabecular bone mineral density (tb.BMD, g/cm^3^), trabecular number (Tb.N, mm/1), and trabecular separation (Tb.Sp, mm) were exported for reconstruction using the manufacturer’s software, Caliper Analyze.

### Hematoxylin-eosin (H&E) staining and histopathological examination

Left proximal tibia joints together with the synovial tissues were fixed in 4% paraformaldehyde and then decalcified in 10% EDTA for approximately 3 weeks. The tissues were embedded in paraffin and stained with H&E for light microscopy analysis. Histopathological quantification was evaluated by three blinded, independent pathologists. Inflammation was scored 0–5 according to the following criteria: 0, normal; 1, minimal inflammatory infiltration; 2, mild infiltration; 3, moderate infiltration with moderate edema; 4, marked infiltration with marked edema; and 5, severe infiltration with edema. Hyperplasia was defined as synovial tissue intimately invading the bone and/or cartilage and was scored 0 to 3 as follows: 0, none; 1, minimal; 2, moderate; and 3, severe. Bone degradation was scored using the following 0 to 3 scales: 0, no bone erosion; 1, mild surface erosion; 2, moderate surface erosion; and 3, strong surface erosion.

### Statistical analysis

Statistical analyses were performed using GraphPad Prism 7 Software (GraphPad Software, Inc., San Diego, CA, USA). The data are expressed as the mean ± standard deviation (SD). For parametric data, comparisons of different groups were performed by one-way analysis of variance, followed by Tukey’s post hoc test for multiple comparisons. *p* <0.05 was considered statistically significant.

## Results

### IPRN ameliorates the inflammatory phenotype of RA FLSs

**Fig. 1:**
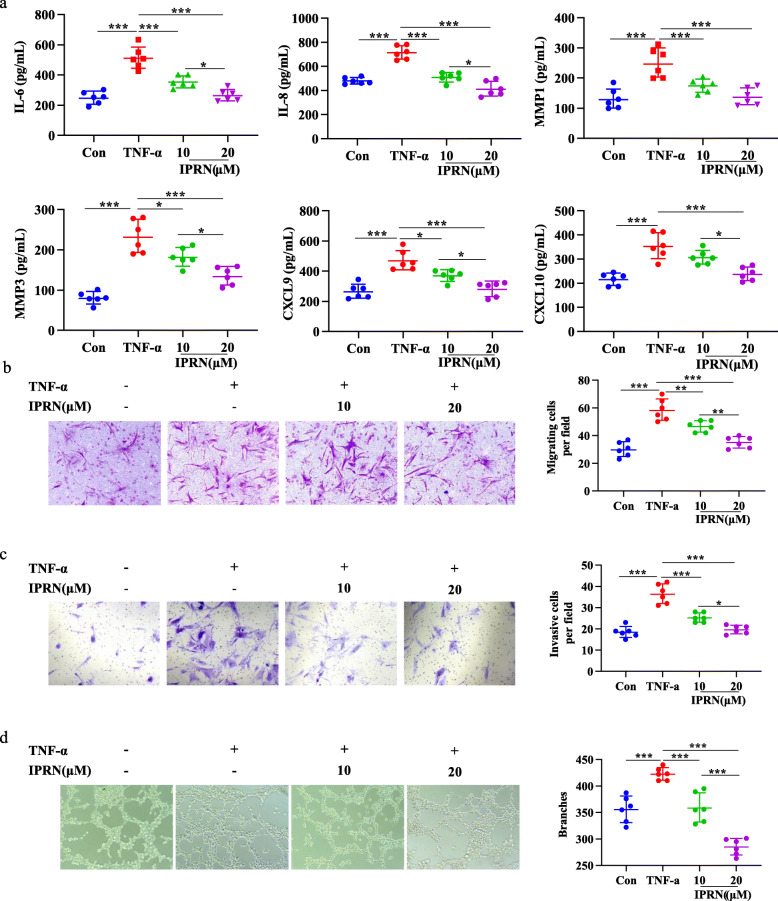
IPRN suppressed the activation of RA FLSs. After treatment with to TNF-α (10 ng/mL), RA FLSs were treated with IPRN (10 μM or 20 μM) or not for 24 h. **a** The expression of IL-6, IL-8, MMP1, MMP3, CXCL9, and CXCL10 were investigated by ELISA. **b**, **c** Cells were collected and Transwell assay were used to evaluate their migration (**b**) and invasion (**c**). **d** Supernatants from RA FLSs were collected and used as conditioned medium to culture HUVECs to perform tube formation assay. The data are expressed as the mean ± SD (*n*=6) and are from three independent experiments with similar results. ^*^P < 0.05, ^**^P < 0.01, and ^***^P< 0.001

RA FLSs display an inflammatory phenotype characterized by the excessive production of inflammatory factors and the enhancement of invasive ability. First, we analyzed the effect of IPRN on cytokine production by RA FLSs exposed to TNF-α. RT-qPCR and ELISA analysis showed that the induction of IL-6, IL-8, MMP1, MMP3, (C-X-C motif) ligand (CXCL)9, and CXCL10 production resulting from TNF-α stimulation could be significantly attenuated by IPRN treatment (Fig. S1, Fig. 1a). Furthermore, following IPRN treatment, the numbers of migrated and invaded RA FLSs notably decreased in a dose-dependent manner (Fig. 1b, c). We also determined the effect of IPRN on the pro-angiogenesis properties of RA FLSs, and the results showed that after exposure to supernatants from IPRN-treated RA FLSs, the angiogenic ability of HUVECs significantly decreased when compared with that of the parental control cells (Fig. 1d). Thus, the above experiments confirmed that IPRN could ameliorate the inflammatory phenotype of RA FLSs.

### IPRN exerts anti-arthritis effects on a CIA model

**Fig. 2:**
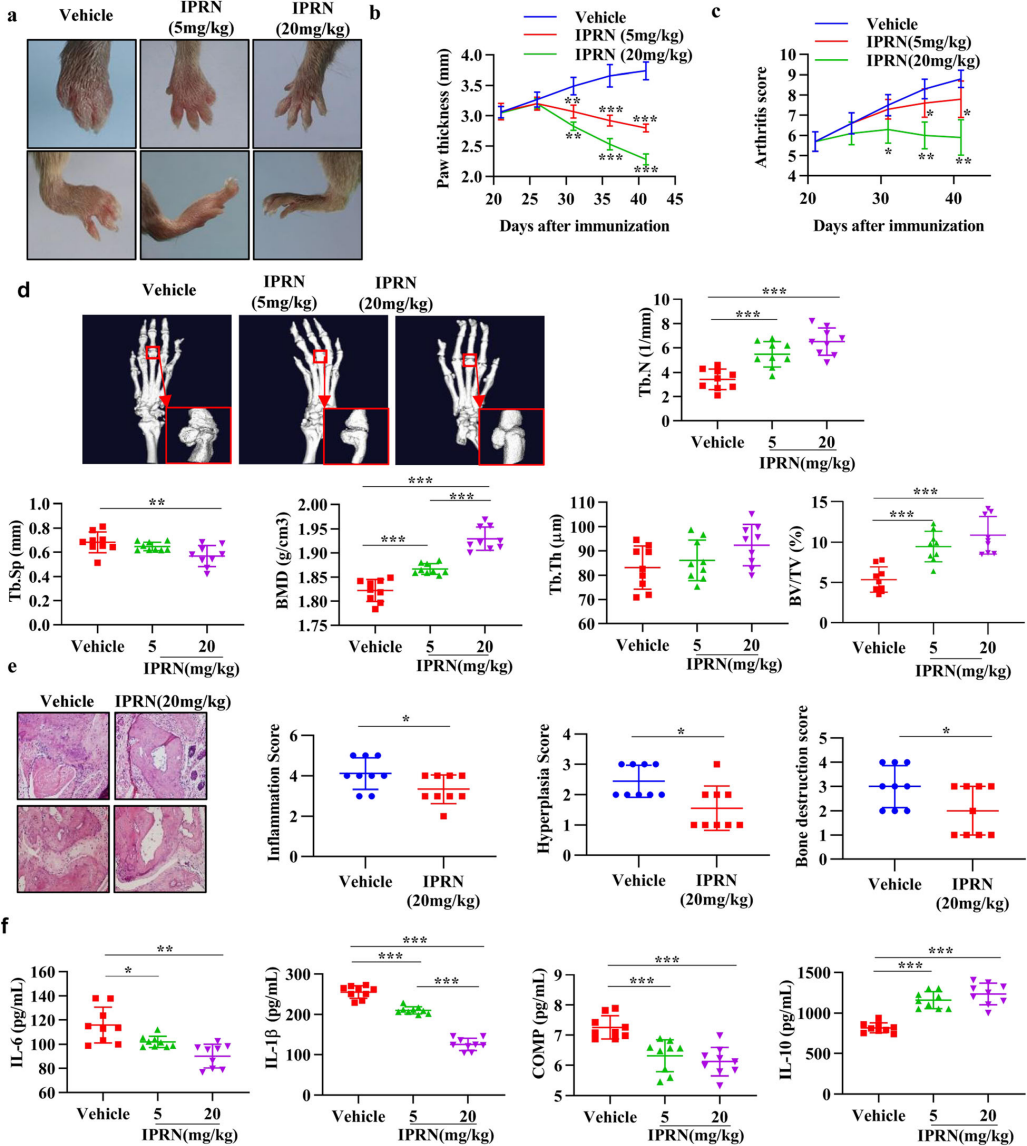
IPRN ameliorated the arthritis phenotype of the CIA model. Mice with CIA (*n*=9) received an intraperitoneal injection of IPRN (5 mg/kg or 20 mg/kg) every 2 days. **a** Representative image of hind paws. **b**, **c** Paw thickness and arthritis score. **d** Micro-CT of ankles. **e** H&E staining and quantification of histological score of ankle samples. **f** The serum levels of IL-6, IL-1β, COMP, and IL-10. The data are expressed as the mean ± SD (*n*=9). ^*^P < 0.05, ^**^P < 0.01, and ^***^P< 0.001

The CIA mouse model was then used to investigate the therapeutic potential of IPRN in RA. The results showed that IPRN could inhibit the pathological progression of the RA-like phenotype, as evidenced by the decreased degree of ankle swelling (Fig. 2a), paw thickness (Fig. 2b), and arthritis score (Fig. 2c), compared to the vehicle treatment. We also used micro-CT to quantify the changes in bone destruction in the mice with CIA. Apparently, the animals in the IPRN-treated group suffered less bone damage in their ankle joints than those in the vehicle-treated group, which was shown by the increase in trabecular BV/TV, BMD, Tb.N, and Tb.Th but the decrease in Tb.Sp (Fig. 2d).

HE staining was then conducted to analyze the effect of IPRN on the pathological characteristics of inflamed joints in mice with CIA treated with or without IPRN. As expected, IPRN ameliorated the pathological characteristics of RA, such as synovial hyperplasia, inflammatory cell infiltration, and cartilage damage (Fig. 2e). The serum levels of proinflammatory cytokines (IL-6 and IL-1β) and anti-inflammatory cytokines (IL-10) were also detected in the mice with CIA. IPRN significantly reduced the production of IL-6, IL-1β, and COMP but increased the production of IL-10 (Fig. 2f). These results further support the potential efficacy of IPRN in RA treatment.

### Prediction of the molecular pathways affected by IPRN in RA FLSs by RNA-Seq

**Fig. 3:**
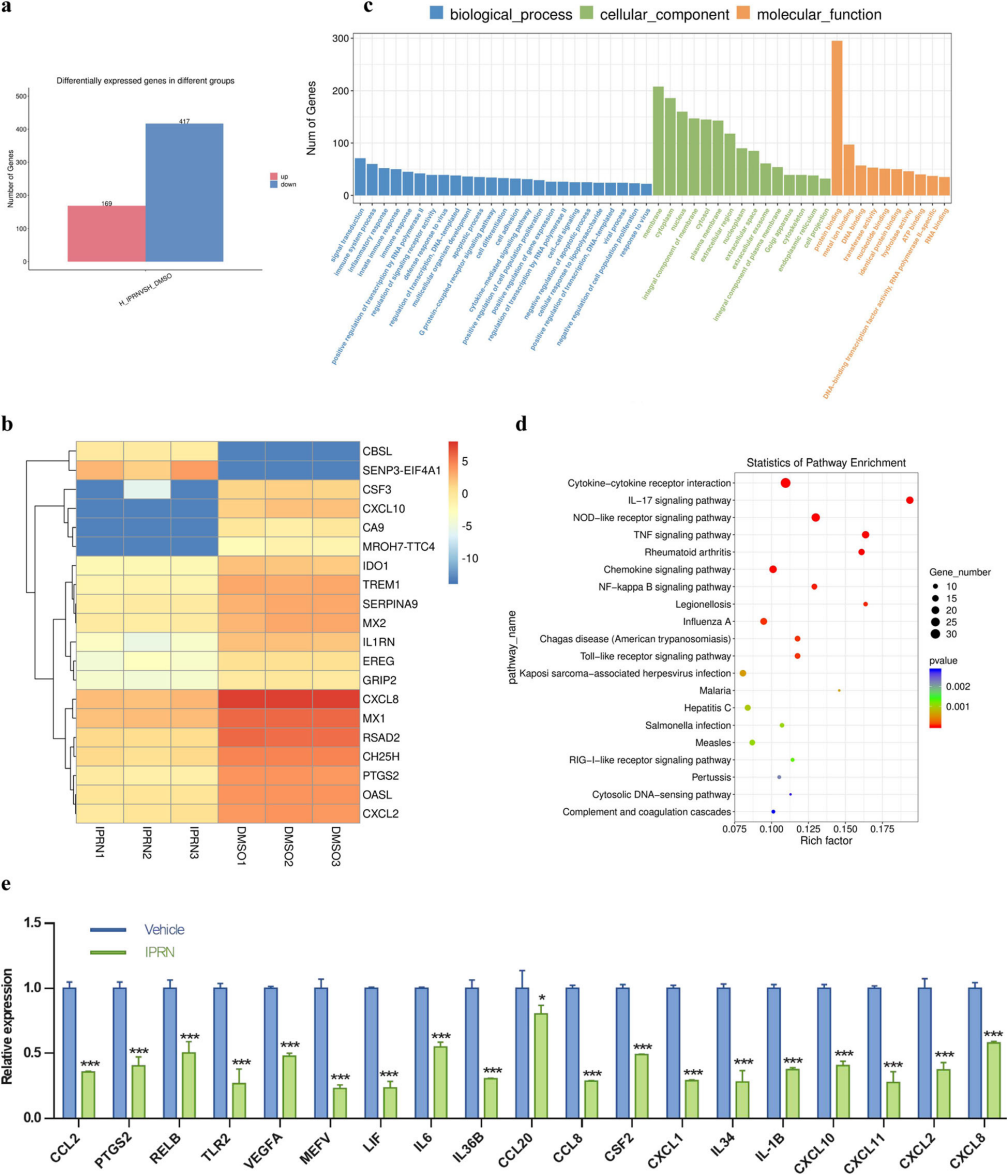
Molecular pathway prediction of the antiarthritic effect of IPRN in RA FLSs by RNA-Seq. RA FLSs were treated without or with IPRN (10 μM) in the presence of TNF-α (10 ng/mL) for 24 h. Total RNA was extracted for RNA-seq. **a** The number of differentially expressed genes (DEGs) induced by IPRN. **b** Heatmap displays different blocks of top 20 DEGs between the IPRN-treated cells (*n*=3) and the control cells (*n*=3). **c** The GO analysis of DEGs induced by IPRN. Bar plots show the significant enrichment terms of the DEGs involving biological process, cellular component, and molecular function ranked by enrichment score (−log10 (*p* value)) values. **d** KEGG enrichment of DEGs. **e** Verification of DEGs by RT-qPCR. The data are expressed as the mean ± SD (*n*=6). ^*^P<0.05, ^***^P < 0.001

To further support the anti-inflammatory role of IPRN in RA, RNA-Seq was performed in RA FLSs treated with or without IPRN. A total of 586 differentially expressed genes (DEGs) were observed when RA FLSs were treated with IPRN compared to the control treatment (Fig. 3a). Among these genes, 169 DEGs were upregulated, while 417 were downregulated. A heatmap of the top 20 DEGs is displayed in Fig. 3b. To acquire a more comprehensive and deep understanding of the DEGs, Gene Ontology (GO) function and Kyoto Encyclopedia of Genes and Genomes (KEGG) pathway enrichment analyses were performed. The annotated results for the GO terms were divided into the biological process (BP), cell component (CC), and molecular function (MF) ontologies (*p* < 0.05, FDR < 0.05) (Fig. 3c). The results of the GO analysis based on BP revealed that DEGs were mainly enriched in signal transduction, immune system process, inflammatory response, and immune response. For the GO analysis focusing on CC, DEGs were significantly enriched in membrane, cytoplasm, nucleus, and integral component of membrane. In addition, the MFs of the DEGs were mainly enriched in protein binding and metal ion binding.

Among the deregulated pathways, the top 5 downregulated KEGG pathways were as follows: cytokine-cytokine receptor interaction (ko04060), IL-17 signaling pathway (ko04657), NOD-like receptor signaling pathway (ko04621), TNF signaling pathway (ko04668), and rheumatoid arthritis (ko05323) (Fig. 3d). Importantly, these pathways play important roles in the progression of inflammatory diseases, including rheumatic diseases [27, 28]. Furthermore, RT-qPCR was used to confirm the expression of DEGs with significant roles in the dysregulated KEGG pathways mentioned above (Fig. 3e), and the results confirmed the reliability of the RNA-seq data. Together, IPRN plays an anti-inflammatory role in RA by suppressing the activities of multiple inflammatory pathways.

### Identifying MIF as IPRN’s direct target

**Fig. 4:**
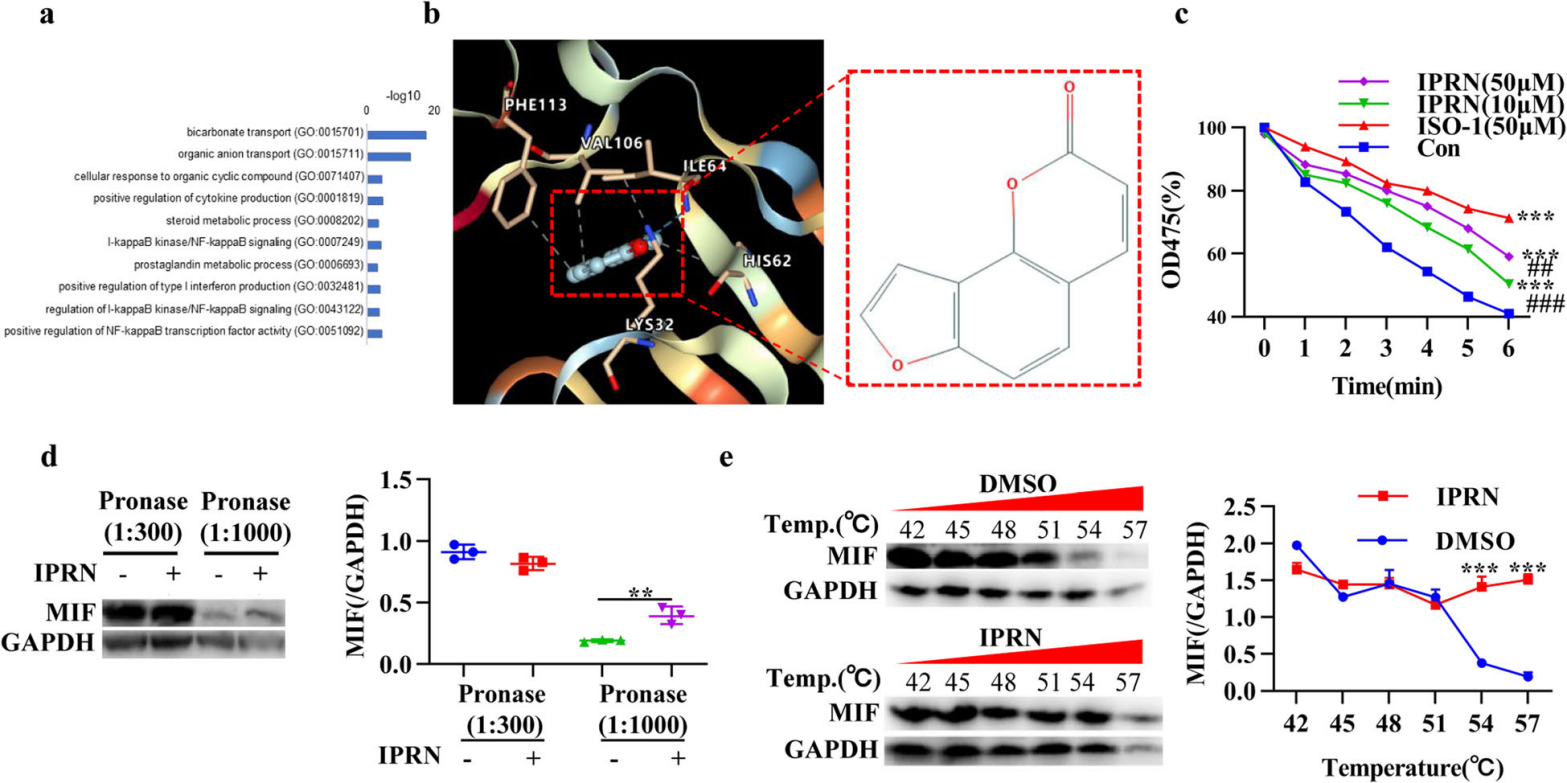
Identification of the targets of IPRN. **a** GO analysis of the potential targets of IPRN identified by network pharmacology analysis. **b** Molecular docking analysis of IPRN and the MIF protein (PDB ID: 1GCZ). **c** Recombinant MIF was incubated with l-dopachrome methyl ester substrate with or without IPRN (10 or 50 μmol/L) or ISO-1 (50 μmol/L). The tautomerase activity was assessed by measuring the absorbance at 475 nm over 6 min. **d** Western blotting analysis of MIF protein expression in RA FLSs after incubation with IPRN. GAPDH was used as a loading control to normalize MIF expression. **e** CETSA assay of the stability of the MIF protein at 54 °C and 57 °C in the absence or presence of IPRN. The values are expressed as the mean ± SD of at least three independent experiments. ^**^P< 0.01 and ^***^P < 0.001

To identify the molecular targets of IPRN, we applied network pharmacology to conduct target prediction analysis. Target prediction was carried out by online tools, such as PharmMapper and STITCH, and a total of 36 targets were identified. GO annotation showed that some targets, such as a disintegrin and metallopeptidase domain (ADAM17), receptor-interacting serine/threonine-protein kinase 2 (RIPK2), Toll-like receptor 9 (TLR9), RAR-related orphan receptor alpha (RORA), MIF, and aldose reductase (AKR1B1), were closely related to inflammation (GO 0001819, GO 0007249, GO 0043122, GO 0051092) (Fig. 4a). Among these inflammation-related targets, ADAM17, TLR9, MIF, nuclear factor kappaB subunit 1 (NFKB1), NF-kappaB subunit (RELA), and Caspase 8 (CASP8) have been shown to promote the disease progression of RA. As MIF was the target with the highest score, we selected MIF for further study.

Human MIF has tautomerase activity, and residues Lys32, Ile64, Val106, and Phe113 are located at the rim of the active sites of the MIF protein [29]. Then, molecular docking of IPRN with MIF (PDB ID:1GCZ) was carried out using DOCK6, and their potential interacting sites are presented in Fig. 4b. Interestingly, IPRN showed a hydrogen bond interaction with residues Lys 32 and Ile 64 of the MIF protein. In addition, IPRN formed hydrophobic interactions with the residues His62, Val 106, and Phe113. We also detected the effect of IPRN on the tautomerase activity of MIF. As expected, IPRN significantly inhibited the enzyme activity of recombinant human MIF (Fig. 4c). Moreover, the activity of MIF in RA FLSs and serum obtained from mice with CIA treated with IPRN decreased (Fig. S2d, e).

To verify the interaction of IPRN with MIF, DARTS and CETSA experiments were conducted. The DARTS results showed that IPRN can protect the degradation of the MIF protein caused by proteases (Fig. 4d). In addition, the CETSA assay showed that IPRN significantly improved the stability of MIF at 54°C and 57°C (Fig. 4e). However, IPRN did not affect the expression of MIF in RA FLSs (Fig. S2a-c).

In conclusion, the results described above indicate that MIF is a potential target of IPRN and that IPRN may target and inactivate MIF.

### IPRN inhibits inflammation via MIF

**Fig. 5:**
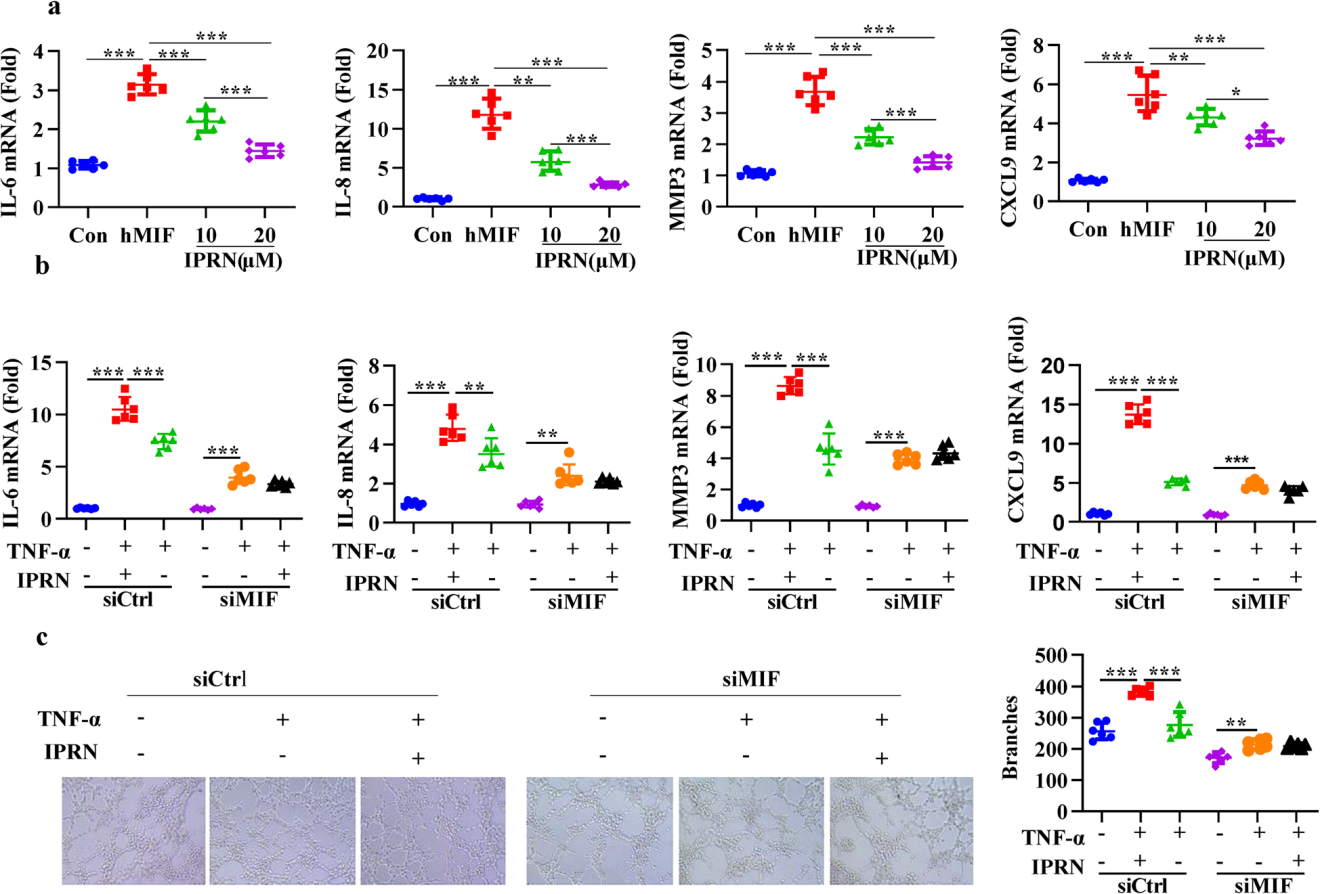
IPRN inhibits inflammation in a MIF-dependent way. **a** In the presence of IPRN (10 μM or 20 μM), RA FLSs were treated hMIF (5 ng/mL) or not for 24 h. Total RNA was extracted and the expression of IL-6, IL-8, MMP3, and CXCL9 at mRNA levels were investigated by RT-qPCR. **b**, **c** RA FLSs transfected with control (siCtrl) or MIF siRNA (siMIF) were stimulated with TNF-α (10 ng/mL). The mRNA expression of IL-6, IL-8, MMP3, and CXCL9 was tested by RT-qPCR (**b**). Supernatants of RA FLSs were collected and used as conditioned medium to culture HUVECs to perform tube formation assays (**c**). The values are expressed as the mean ± SD (*n*=6) and are from three independent experiments with similar results. ^*^P < 0.05, ^**^P < 0.01, and ^***^P< 0.001

MIF acts as an important contributor to the pathological progression of RA. Consistently, following treatment with recombinant human MIF, the expression of IL-6, IL-8, MMP3, and CXCL9 significantly increased. However, this increase could be attenuated when RA FLSs were simultaneously treated with IPRN (Fig. 5a). To further confirm the importance of MIF for the anti-inflammatory effect of IPRN, siRNA targeting MIF was introduced. After knocking down MIF expression in RAFLSs, the inhibitory effects of IPRN on TNF-α-induced cytokine release (Fig. 5b) and the pro-angiogenic ability (Fig. 5c) of RA FLSs could hardly be observed. These data suggest that MIF acts as an essential contributor to the anti-inflammatory effect of IPRN.

## Discussion

The application of disease-modifying anti-rheumatic drugs (DMARDs) and biological agents has improved the therapeutic effects in RA [30]. However, these treatment strategies still have some problems, especially the increased susceptibility to infection caused by long-term treatment or the existence of refractory rheumatoid arthritis patients who do not respond well to these existing medicines [31, 32]. Recently, complementary and alternative medicine approaches including TCM have been shown to increase the likelihood of complete remission of RA [32, 33]. More detailed studies concerning the antiarthritic mechanism of TCMs or their natural ingredients may provide novel treatment strategies for RA.

Previous studies on IPRN have mainly focused on its antibacterial, osteogenic, and anti-inflammatory effects. Following treatment of ovalbumin-induced lung injury models with IPRN, the infiltration of inflammatory cells and the production of inflammatory factors were suppressed [34]. Furthermore, IPRN prevented the alveolar bone loss and obviously inactivated the inflammatory response in mice with periodontitis [15]. Mechanistically, IPRN protects against the inflammation caused by lipopolysaccharide (LPS) challenge in both in vitro and in vivo models by inhibiting the activation of the NF-κB and mitogen-activated protein kinase (MAPK) pathways [35]. In this study, we characterized the antiarthritic effect of IPRN in both cultured human RA FLSs and a CIA model, and IPRN represents a potential drug for RA treatment that can inhibit the inflammatory phenotype of RA FLSs and prevent the onset and progression of RA-like manifestations in a CIA model.

MIF acts as a pleiotropic inflammatory cytokine that is important in both innate and adaptive immunological responses and has been implicated in the pathogenesis of autoimmune diseases, including RA and systemic lupus erythematosus (SLE) [36]. Elevated levels of MIF expression were observed in synovial tissues and synovial fluids from RA patients compared to osteoarthritis (OA) patients or healthy volunteers [37]. Specifically, the expression of MIF was closely related to the activity of RA, and its expression at higher levels indicated more severe bone erosion [38]. Functionally, MIF was required for sustaining inflammatory responses by inducing the production of proinflammatory cytokines and tissue-degrading molecules, promoting the proliferation and survival of synovial fibroblasts, stimulating the chemotaxis of neutrophils, and increasing angiogenesis and osteoclast differentiation. In animal models of RA, the genetic and therapeutic inhibition of MIF has been shown to suppress inflammation and ameliorate bone destruction [39, 40]. Furthermore, MIF-targeted treatments are being examined in preclinical studies or early-stage clinical trials due to their specific functions observed in different autoimmune diseases. The development of small-molecule MIF antagonists could provide a means of selectively intervening in the pathogenesis of RA [40, 41]. Here, our data confirmed that IPRN targeted and inactivated MIF and that MIF was required for the anti-inflammatory effect of IPRN, further confirming the likelihood that IPRN could be further exploited as a drug for RA treatment.

## Conclusion

In summary, the results from our in vivo and in vitro experiments support the antiarthritic effects of IPRN in RA, and MIF represents a target by which IPRN exerts its anti-inflammatory effects. Therefore, IPRN holds great potential as a novel therapeutic agent for RA.

## Supplementary Information

**Additional file 1: Figure S1.**

**Additional file 2: Figure S2.**

**Additional file 3: Supplied Table 1.**

## Data Availability

The authors are committed to sharing their data, publishing the data, and making available the resources described in this publication to the scientific community.

## Abbreviations

RA: Rheumatoid arthritis
micro-CT: Computed tomography
TNF-α: Tumor necrosis factor-α
ILs: Interleukins
FLSs: fibroblast-like synoviocytes
MMP: Matrix metalloproteinase
TCM: Traditional Chinese medicine
IPRN: Isopsoralen
CIA: Collagen-induced arthritis
MIF: Macrophage migration inhibitory factor
GAPDH: Glyceraldehyde-3-phosphate dehydrogenase
TBST: Tris-buffered saline with tween
RNA-Seq: RNA sequencing
DARTS: Drug affinity responsive target stability
CETSA: Cellular thermal shift assay
CXCL: (C–X–C motif) ligand
BV/TV: Bone volume/total volume
BMD: Bone mineral density
Tb.N: Trabecular number
Tb.Th: Trabecular thickness
Tb.Sp: Trabecular separation
COMP: Cartilage oligomeric matrix protein
DEGs: Differentially expressed genes
GO: Gene ontology
KEGG: Kyoto Encyclopedia of Genes and Genomes
ADAM17: A disintegrin and metallopeptidase domain 17
RIPK2: Receptor-interacting serine/threonine-protein kinase 2
TLR9: Toll-like receptor 9
RORA: RAR-related orphan receptor alpha
AKR1B1: Aldose reductase
DMARDs: Disease-modifying anti-rheumatic drugs
NF-κB: Nuclear factor-kappaB
MAPK: Mitogen-activated protein kinase
NFKB1: Nuclear factor kappaB subunit 1
RELA: NF-kappaB subunit
CASP8: Caspase 8
