# Supplementary figures and images for: Isopsoralen ameliorates rheumatoid arthritis by targeting MIF
Source: Arthritis Res Ther. 2021 Sep 17;23:243. doi: 10.1186/s13075-021-02619-3 (PMC8447788; doi:10.1186/s13075-021-02619-3)

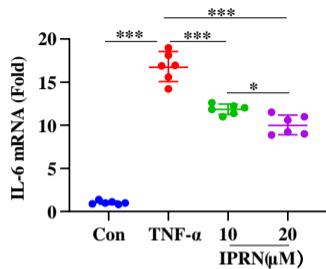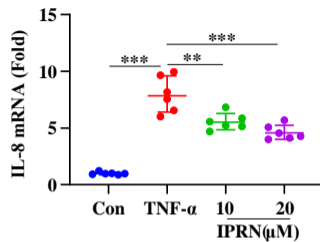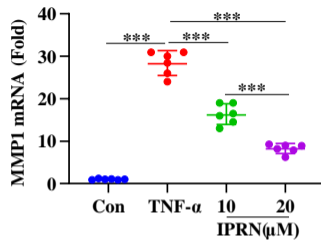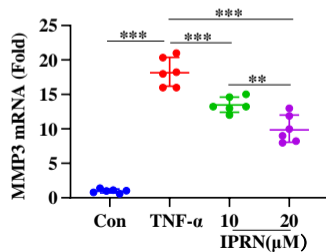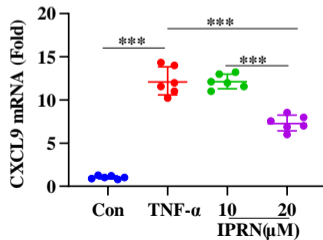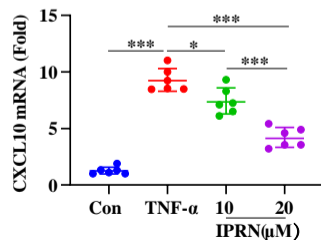

Supplement: Supplementary file 1 — Additional file 1: Figure S1. [file 13075_2021_2619_MOESM1_ESM.pdf]

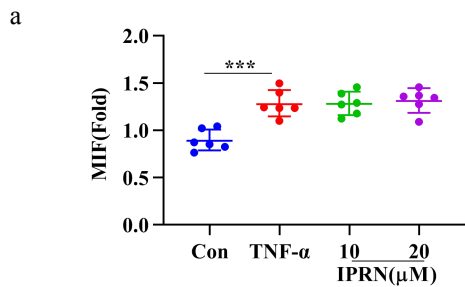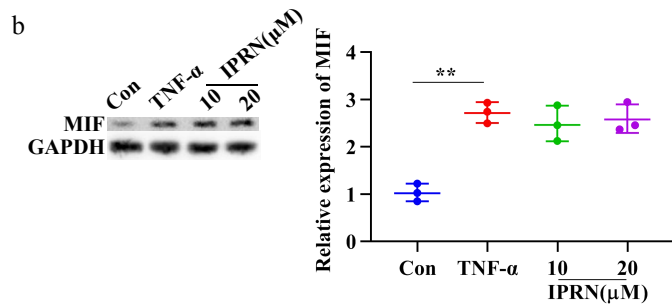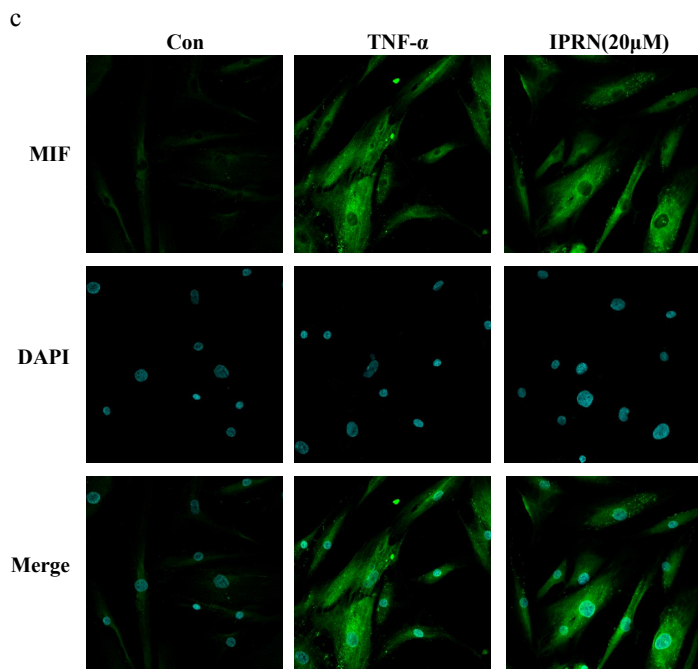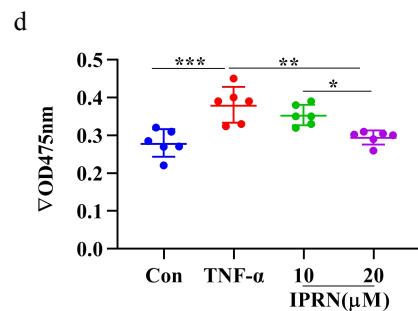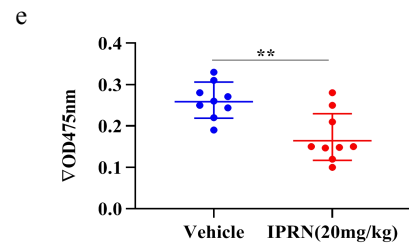

Supplement: Supplementary file 2 — Additional file 2: Figure S2. [file 13075_2021_2619_MOESM2_ESM.pdf]
