# Supplementary material for: Isopsoralen ameliorates rheumatoid arthritis by targeting MIF
Source: Arthritis Res Ther. 2021 Sep 17;23:243. doi: 10.1186/s13075-021-02619-3 (PMC8447788; doi:10.1186/s13075-021-02619-3)
Supplement: Supplementary file 3 — Additional file 3: Supplied Table 1. [file 13075_2021_2619_MOESM3_ESM.docx]

| Gene | Forward primer | Reverse primer |
| --- | --- | --- |
| IL-6 | CCACCGGGAACGAAAGAGAA | GAGAAGGCAACTGGACCGAA |
| IL-8 | CAGTTTTGCCAAGGAGTGCTAA | AACTTCTCCACAACCCTCTGC |
| MMP1 | TGTTCTGGGGTGTGGTGTCT | CTGAGCCACATCAGGCACTC |
| MMP3 | CTGGACTCCGACACTCTGGA | CAGGAAAGGTTCTGAAGTGACC |
| MIF | CCACGGGGATTGTCAGTGAAG | CTGTGCAGGTTTGTCTGTTCC |
| CXCL9 | CCAGTAGTGAGAAAGGGTCGC | AGGGCTTGGGGCAAATTGTT |
| CXCL10 | GTGGCATTCAAGGAGTACCTC | TGATGGCCTTCGATTCTGGATT |
| GAPDH | TGATGACATCAAGAAGGTGG | TTACTCCTTGGAGGCCATGT |
| CCL2 | AGAGGCTGAGACTAACCCAGA | TTTCATGCTGGAGGCGAGAG |
| PTGS2 | TAAGTGCGATTGTACCCGGAC | TTTGTAGCCATAGTCAGCATTGT |
| RELB | CAGCCTCGTGGGGAAAGAC | GCCCAGGTTGTTAAAACTGTGC |
| TLR2 | ATCCTCCAATCAGGCTTCTCT | GGACAGGTCAAGGCTTTTTACA |
| VEGFA | AGGGCAGAATCATCACGAAGT | AGGGTCTCGATTGGATGGCA |
| MEFV | TAAGACCCCTAGTGACCATCTG | TTCCCCATAGTAGGTGACCAG |
| LIF | CAAGCGCCGTATGGGACTTT | GGAGGCATCCATGTAGCTCT |
| IL36B | ATGAACCCACAACGGGAGG | TAATGCTGCGGCTAAGAGGAG |
| CCL20 | GCTGTACCAAGAGTTTGCTC | AGTTGCTTGCTTCTGATTCG |
| CCL8 | TGGAGAGCTACACAAGAATCACC | TGGTCCAGATGCTTCATGGAA |
| CSF2 | GCTGTCTACGTCGGGATGC | GACCATGCGATCCACCTCTC |
| CXCL1 | TGCTGCCACTAATGCTGATGT | CTCAGGAACCAATCTTTGCACT |
| IL34 | AAGGTGGAATCCGTGTTGTCC | AGCTTTGTTTACAGCAGGAGC |
| IL-1B | ATGATGGCTTATTACAGTGGCAA | GTCGGAGATTCGTAGCTGGA |
| CXCL11 | CAGTTGTTCAAGGCTTCCC | ATCTGCCACTTTCACTGCT |
| CXCL2 | CTCAAGAACATCCAAAGTGTG | ATTCTTGAGTGTGGCTATGAC |
| CXCL8 | ACTCCAAACCTTTCCACCC | CAATAATTTCTGTGTTGGCGC |

**Supplement table 1** | Sequence of primers used in the present study
